# Malaria, gastrointestinal parasite infection and nutritional status among febrile children In Accra, Ghana

**DOI:** 10.21203/rs.3.rs-2891006/v1

**Published:** 2023-05-08

**Authors:** Bright Amoah Darko, Christopher Mfum Owusu-Asenso, Kantanka Addo-Osafo, Edith Appiah-Lawson, Yaw Asare Afrane, Edem Magdalene Afua Tette

**Affiliations:** Department of Medical Microbiology, University of Ghana Medical School; Department of Medical Microbiology, University of Ghana Medical School; Department of Medical Microbiology, University of Ghana Medical School; Department of Medical Microbiology, University of Ghana Medical School; Department of Medical Microbiology, University of Ghana Medical School; Department of Community Health, University of Ghana Medical School

**Keywords:** Malnutrition, Stunting, Wasting, Anaemia, Febrile, Underweight

## Abstract

**Introduction:**

Malaria and intestinal parasite infection are common in developing countries. These Parasites causes anaemia and malnutrition mostly in children. For this reason, it is important to study these infections and their effects in order to monitor interventions to control them. This study aims to determine prevalence of malaria and intestinal parasite infections and their association with nutritional status among febrile children in Accra, Ghana.

**Methods:**

The study was conducted among febrile children aged 6 months to 5 years attending three health facilities in Accra from May to October, 2022. A total of 315 children were selected for the study. Anthropometric measurement was done for each participant. Blood and stool samples were collected for investigation. Thick and thin blood smears stained with 10% Giemsa were prepared and examined for Plasmodium parasite using microscopy. Stool samples were processed using direct wet mount and formalin-ether concentration method and examined for intestinal parasites using microscopy. Haemoglobin concentration was measured using automatic haematology analyzer.

**Results:**

A total of 24% (76/315) were positive for malaria. *Plasmodium falciparum* accounted for 77.6% (59/76) of parasitaemia, whereas *Plasmodium malariae* was 22.4% (17/76). Prevalence of intestinal parasite infection was 10.7% (34/315). *Giardia lamblia* accounted for 17/315 (5.3%) of the entire children, followed by *Ascaris lumbricoides* 8/315 (2.5%), Hookworm 6/315 (1.9%) and *Trichuris trichiura* 3/315 (0.9%). A total of 15/315 (5%) of the participants had co-infection of malaria and intestinal parasite infection. Prevalence of anaemia, malnutrition, stunting, wasting and underweight were (72%), (30.7%), (16.2%), (24.4%) and (57.1%) respectively. Malaria was significantly associated with anaemia (p = 0.000) and underweight (p = 0.013). Ascaris lumbricoides was significantly associated with wasting (p = 0.010). Giardia lamblia was significantly association with malnutrition (p = 0.000) and Stunting (p = 0.000), whereas Hookworm was found to be significantly associated with anaemia (p = 0.021).

**Conclusion:**

Prevalence of IPI in this study was less than previously reported, most likely due to regular deworming of most of the children. However, Malaria and intestinal parasitic infection were significantly associated with anaemia and malnutrition including wasting, stunting, and underweight.

## Background

Malaria and intestinal parasite infection (IPI) are of great public health concern, causing death and morbidity in South-Saharan Africa [1, 2]. Recent reports indicates that, about 627,000 and 200,000 cases of mortality due to malaria and intestinal parasite infection respectively have been recorded worldwide, with majority from West Africa [3, 4]. In Ghana, these parasites are common and affect a large number of people each year [5–8]. Due to the high prevalence of malaria and IPI, these conditions have been listed among the top five outpatient diseases in the country [7]. Furthermore, the most prevalent and severe form of malaria is caused by the parasite *Plasmodium falciparum*. *Plasmodium falciparum* accounts for more than 90% of malaria mortality in Ghana [9, 10]. High morbidity has been associated with children under the age of five due to their weak immune system [11–13].

Undernutrition in children is reported to be an important public health issue in Ghana [14]. Nutritional status is closely linked to immune function and infectious disease [15]. A study by Black et al., indicated that children who are malnourished can develop a number of diseases [16]. Another study conducted in the northern part of Ghana reported that malnutrition in children is a fundamental factor contributing to malaria-associated morbidity in Ghana [17]. Since malnutrition reduces immunity to infection, malnutrition makes people vulnerable to malaria [18]. Malaria reduces haemoglobin levels through enhanced destruction of parasitized red blood cells (RBCs), which results in anaemia [19, 20]. *Plasmodium falciparum* infection causes loss of appetite, which might lead to underweight [21].

Intestinal parasite infections of public health importance in Ghana are amoebiasis, giardiasis, acariasis, hookworm, and trichuriasis [22, 23, 7]. These parasites can potentially cause nutritional deficiencies [24]. *Ascaris lumbricoides* has the potential to bind to the absorptive surfaces of the intestinal walls and affect intake of nutrients meant for the body of its host, leading to malnutrition [24, 25]. Hookworm infection and trichuriasis can cause chronic blood loss, resulting in iron deficiency anaemia, whereas giardiasis and amoebiasis may cause diarrhea and malabsorption leading to stunted growth [26]. To prevent this, the Child Health Program (CHP) in Ghana recommends regular deworming of children, and this has been integrated into services delivered at child welfare clinics. However, this practice is actioned by individual parents rather than through mass treatment [27]. Thus, the effect of the intervention needs to be monitored, particularly as there have been concerns about the effectiveness of the medication used in deworming children in other parts of the world [28, 29].

In Ghana, studies have established the endemicity of malaria and intestinal parasitic infections [6, 30, 31]. However, research on the impact of these parasitic infection on nutritional status among children is limited [23]. There is a need to investigate the association of malnutrition and parasitic infections occurring in children. This will help provide information to aid policy on the treatment of these infections. Therefore, this study sought to determine the prevalence of malaria and intestinal parasite infection and their association with nutritional status among febrile children in Accra.

## Materials and methods

### Study design

This study was a cross-sectional study carried out at the outpatient department (OPD) and inpatient department (emergency and admission wards) of three hospitals in Accra from May 2022 to October 2022. A semi-structured questionnaire was used to collect data. Anthropometry was also measured.

### Study area

This study was conducted at three hospital facilities in Accra: Princess Marie Louise Children Hospital, Shukura Hospital, and Mamprobi Hospital. Princess Marie Louise Children’s hospital (PML) (5.5488° N, 0.2116° W) is located within Ashiedu Keteke sub-metropolitan district with a population of 443,768 people [32]. The hospital offers both secondary and primary care for paediatric patients from birth to 18 years old. It has 74 bed capacity and the largest nutritional rehabilitation centre in the country [14].

Shukura community hospital (5.5488° N, 0.2501 ° W) is one of the best medical facilities located in the Ablekuma Central Municipality in Accra. Shukura Community hospital provides medical services for people in the municipality. It is a 61-bed capacity hospital and patients are mostly self-referred. Mamprobi hospital (5.5367° N, 0.2421 °W) is located in the Ablekuma South Sub Metropolitan District in Accra. The population of Ablekuma South District is 15,051 [32]. It has 54 bed capacity and over 250 patients visit per day. It offers clinical and healthcare services, including disease treatment, reproductive health, child health and nutrition to people living in the municipality.

### Study population

Febrile children (body temperature > 37.5°c) with diarrhea, from the age of 6 months to 5 years old who reported to the hospital were eligible for this study. Febrile children who had convulsion based on evidence from the medical history were excluded from the study.

### Sampling size determination

The minimum sample size for the study was determined using Cochran’s formula, n = Z ^2^pq/e^2^. Using a 95% confidence level, 0.5 standard deviations, and ± 5% precision; where n = Minimum sample size, z = Z Score (at a 90% confidence level, Z score = 1.645), p = An estimated proportion of an infection that is present in the population (50%), e = percentage margin of error taken as 5% and q = 1-p (Pourhoseingholi et al., 2013). Thus, n = Z^2^pq/e^2^ = (1.645)^2^(0.5) (0.5/ (0.05)^2^ = 278. Therefore, the minimum sample size (n) for this study was 278.

### Sampling method

Participants were selected from each study sites using a convenient sampling method. Selection of this method was based on study participants availability at the selected hospitals.

### Data collection

In-person interviews were used to collect primary data using a semi-structured questionnaire with questions about participant’s sociodemographic characteristics such as gender and age, and medical history such as clinical signs and symptoms. Weight was measured with a calibrated Seca 750 Robust mechanical floor scale to the nearest 0.1Kg for children above 2 years at all study sites. Weight was measured with a calibrated Seca 374 scale for children below 2 years at all study sites. Height measurement was taken with Seca 213 stadiometer to the nearest 0.1cm at PML and Mamprobi Hospital, and Seca 216 stadiometer to the nearest 0.1cm at Shukura Hospital for children above 2-year-old. Length was taken to the nearest 0.1cm with Seca 416 baby infantometer at PML Hospital, and Seca 210 at Mamprobi and Shukura Hospital for children below 2 years. Mid-upper arm circumference was measured with MUAC tape to the nearest 0.1cm and recorded using World Health Organization (WHO) standard [33]. The anthropometric indices: height-for-age (HA), weight-for-age (WA), weight-for-height (WH) were expressed as Z-scores using the WHO child growth standard [34]. Measurement was done by the principal investigator and assisted by trained nurses using standard techniques.

### Sample collection and examinations

Blood and stool samples were collected from the participants. About 3ml of a venous blood sample was collected into test tubes containing EDTA anticoagulant using aseptic techniques for haemoglobin (Hb) measurements and malaria testing. Parents/guardians were instructed to put a teaspoon of the participant’s stool into the clean, leak-proof wide-neck stool containers provided. Stool samples were processed within 6 hours of collection and examined microscopically within 30 minutes of preparation. Both direct wet mount and formol-ether concentration technique was used to confirm the presence of intestinal parasites in stool [35].

Thin and thick blood smears were prepared, stained with 10% Giemsa, and observed under a light microscope using 40x and 100x objective lenses for malaria parasites. Malaria parasite density was calculated by comparing number of parasites against 200 leucocytes in thick films [36]. Heamoglobin concentration was measured using automatic haematology analyzer (Mindray, Nanshan, Shenzhen, China).

### Data processing and analysis

Data from interviews and parasitic investigations were entered into Microsoft Excel 2016, and exported into Statistical Package for Social Sciences (SPSS, version 26) for statistical analyses. Anaemia was defined as Hb < 11 g/dl; severe anaemia, Hb < 7 g/dl; moderate anaemia, Hb 7 to < 10 g/dl; and mild anaemia, Hb 10–11 g/dl [37]. Children were classified as stunted, underweight or wasted when their HA, WA or WH Z-scores were <−2 below the reference mean, respectively (WHO, 2006). A child was identified as being malnourished with a Mid upper arm circumference (MUAC) measurement < 13.5cm. Moderate malnutrition was identified as MUAC < 12.5-11.5cm, whiles severe malnutrition was classified as MUAC < 11.5cm [33]. Descriptive statistics (frequencies and percentages) were used to determine the prevalence of parasitic infections. Association between parasitic infections and nutritional status was determined using chi square test and univariate logistic regression analysis and their crude odd ratio (95% confidence interval) was reported. Multiple logistic regression models were run for statistically significant variables to determine the significant risk factors. Statistical significance threshold was set at p ≤ 0.05.

## Results

### Socio-demographic and medical history of study participants

Overall, 315 febrile children were enrolled in this study. A total of 113/315 (35.9%) study participants were recruited from Princess Marie Louise Children’s hospital, 100/315 (31.7%) from Mamprobi hospital and 102/315 (32.4%) from Shukura hospital. Out of the total study participants recruited for the study, 182 (58%) were female and 133 (42%) were male. Mean (SD) of the age(s) of participants was 1.17 (± 0.691). The average mean (SD) of the weight and height of participants were 11.95 (± 3.479) kg and 86.6 (± 17.535) cm respectively. Among the clinical presentations of the participants were headache (29.5%), vomiting (34.6%) and chills (35.9%). The socio-demographic characteristics of the participants have been summarised in Table 1.

#### Prevalence of Parasitic infection among study participants.

**Figure 1:**
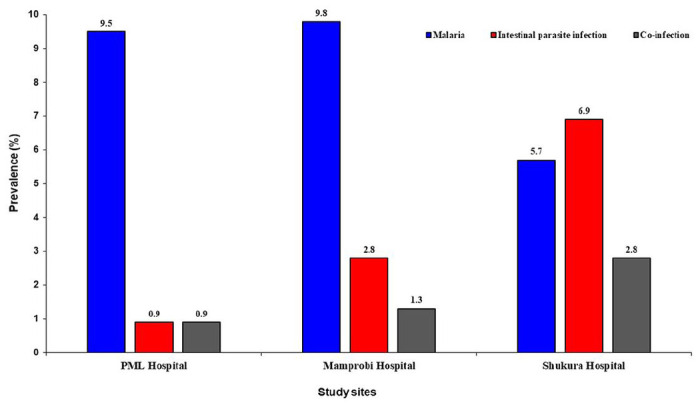
Prevalence of parasitic infection at study sites.

Out of 315 participants, a total of 24% (n = 76/315; 95% CI 21.3–27.8) were positive for malaria, of which *Plasmodium falciparum* constituted 77.6% (59/76) of parasitaemia, whereas *Plasmodium malariae* was 22.4% (17/76) (Table 1). Malaria cases were common among febrile children at Mamprobi hospital 9.8% (31/315) compared to PML hospital 9.5% (30/315) and Shukura hospital 5.7% (18/315) (Fig. 1). Malaria was more prevalent in female children 13.6% (43/315) than in male children 10.4% (33/315). However, there was no association between malaria and gender (χ^2^ = 1.431, df = 2, p = 0.731) (Table 2). Majority of the children between 1–3 years had more malaria prevalence 11.7% (37/315) compared to other age group(s). However, malaria was not significantly associated with age (χ^2^ = 2.285, df = 2, p = 0.319) (Table 2). In this study, 10.8% (n = 34/315; 95% CI 6.4–14.2) of the febrile children were positive for intestinal parasite infection. *Giardia lamblia* was the most recorded intestinal parasite species (5.3%) (Table 1). Among the various study sites, the majority of participants at PML hospital (51%) had used anti-helminth medications in the last three months before the study compared to children at Shukura hospital (47%) and Mamprobi hospital (44%). However, children at Shukura hospital recorded more intestinal parasite infection (6.9%), followed by Mamprobi hospital (2.8%) and PML hospital (0.9%) of the total participants (Fig. 1). Female children were more infected with intestinal parasites (7.3%: 23/315) than their male counterparts (3.4%: 11/315). However, no significant difference was found between intestinal parasite infection and gender (χ^2^ = 0.493, df = 3, p = 0.920) (Table 2). The prevalence of intestinal parasite infection was common in children between 4-5years (5.7%: 18/315). However, the difference observed between intestinal parasite infection and age was not statistically significant (χ^2^ = 7.083, df = 6, *p* = 0.313) (Table 2). About 5% (n = 15/315; 95% CI 2.7–9.1) of the children were co-infected with malaria and intestinal parasites (Table 1).

#### Anaemia, malnutrition, and their relationship to parasite infection.

The average haemoglobin (Hb) mean value (± SD) was 10.420 (± 1.165). A high prevalence of anaemia was recorded among the study participants 72% (n = 227/315; 95% CI 61.3–84.9) (Table 1). Anaemia was significantly associated with being female children (χ^2^ = 0.164, df = 1, p = 0.003) (Table 3). Children between 1–3 years had an increased prevalence of anaemia (34.9%) compared to other age groups. However, there was no significance association between anaemia and age (Table 3). A significant association was observed between children infected with *Plasmodium falciparum* and anaemia (χ^2^ = 1.45, df = 2, p = 0.000) (Table 3). Out of the total participants 34/315 (10.7%) that were infected with intestinal parasite, 32.2% (11/34) also had anaemia. Chi square analysis revealed a significant association between hookworm infection and anaemia (χ^2^ = 1.3, df = 2, p = 0.000) (Table 3). Furthermore, more than half of the children with malaria and co-infection with intestinal parasite co-infection were anaemic (Table 3). A total of 30.7% (n = 96/315; 95% CI 28.3–36.4) of the febrile children were malnourished (Table 1). The prevalence of the various forms of malnutrition including stunting, wasting and underweight were also recorded among the study participants (Table 1). Malnutrition was common among female children (18.4%) in comparison to males (12.3%). However, there was no significant association between malnutrition and gender (Table 3). Malnutrition was significantly associated with children between 6-11 months old (80/227. (χ^2^ = 3.701, df = 2, *p* = 0.003) (Table 3). Over 30% (23/76) of study participants infected with malaria were malnourished whereas 23.5% (8/34) of children with IPI were malnourished. There was no significant association between malaria and malnutrition (Table 3). Nevertheless, a significant association was observed between G*iardia lamblia* and malnutrition (χ*2* = 4.302, df = 2, *p* = 0.000) (Table 3). Prevalence of stunting, wasting and underweight among febrile children in relation to gender and age are summarized in Table 4. A significant association was found between malaria caused by *Plasmodium falciparum* and underweight (χ2 = 2.082, df = 1, *p* = 0.013) (Table 4). Intestinal parasite infection caused by *Giardia lamblia* and *Ascaris lumbricoides* were significantly associated with stunting (χ^*2*^ = 3.412, df = 3, p = 0.000) and wasting (χ2 = 4.052, df = 3, *p* = 0.010) respectively as shown in table Table 4.

## Discussion

Healthy growth, especially in children, is reflected in their nutritional status. Studies show that, there is a strong link between nutritional status and parasitic infection [38]. A child with nutritional deficiency has an increased susceptibility to parasitic infection [39]. At the same time, children infected with parasitic infection are more likely to have nutritional deficiency [40]. This study investigated the prevalence of malaria, intestinal parasite infection and their association with nutritional status among febrile children in Accra.

Findings from this study indicated a high malaria prevalence (24%). However, this finding was contrary to a study by Kanwugu and colleagues in Tamale Metropolis, Ghana. They reported a lower malaria prevalence (2.6%) [41]. These differences could be due to the fact that the present study was hospital based and only febrile children were enrolled to participate in the study compared to theirs which was a community-based study and healthy children were included. Malaria cases were lower in children under one year, which could be due to passive transfer of maternal antibodies through the placenta [42]. A study by Journal et al., [43], also found that malaria occurred more frequently in older children aged 3years old compared to younger children of one year old.

In this study, a lower prevalence of intestinal parasite infection was observed (10.7%). These findings differ from a study by Martinez and colleagues in overcrowded urban slums in Accra, Ghana, who reported a higher prevalence of IPI (47%) [44]. Our prevalence of 10.7% is lower than 17.3% reported by Mirisho et al. [23] at PML hospital in Accra. Another study by Gelaw and colleagues in Northwest Ethiopia, reported a higher prevalence of intestinal parasites (34.2%) [45]. The low IPI prevalence reported in our study, may have been due to the nationwide policy of regular deworming of children by the Ghana Health Service which could account for these disparities [45].

In this study, *Giardia lamblia* was the most prevalent intestinal parasite species. This result is consistent with a research study carried out by Anim-Baidoo et al [11]. They reported that the most dominant parasite was *Giardia lamblia* species (5%) of all children in their study at Princess Marie Louise Children’s Hospital in Accra, Ghana. They emphasized that, assemblage B could be the predominant genotype causing giardiasis in children. Intestinal parasite infection was common among febrile children at Shukura hospital in this study. This could be due to the poor sanitation and hygiene within the community [47]. Lack of proper and clean sanitation facilities promotes open defecation practices such in the rivers and bushes which can directly contaminate water and soil.

Findings from this study revealed that more than 70% of the children were aneamic. This result is higher than 53.8% reported in Hohoe Municipality in Ghana [48] and 49.2% in Enugu, Nigeria [49]. This study provides evidence that there was an association between anaemia and age which aligns with other findings [50, 51], but in contrast to a studies by Id & Jibril [52]. In the current study, species-specific analysis revealed a significant association between different parasite species and anaemia. *Plasmodium falciparum* and hookworm were significantly associated with anaemia. This finding corroborates with a study by Osazuwa et al. [53] in Nigeria. They reported an association between infection with multiple parasites and anaemia, as was shown by the high prevalence of anaemia with co-infection.

The prevalence of malnutrition in this study was 30.7%, which raises concerns for public health. Prevalence of stunting (16.2%), wasting (24.4%), and underweight (74%) among febrile children in this study emphasizes the extent of growth retardation in the studied area. *Giardia lamblia* was significantly associated with malnutrition in this study. This finding agrees with what was earlier reported by Kanokwanvimol et al. [54].

Findings of the current study revealed a significant association between malaria and underweight. This evidence is stronger than an earlier study conducted in Ethiopia by Hassan and Ali, [55]. Their study reported that severely underweight children had an increased risk of a clinical malaria attack, but this finding was not statistically significant. Intestinal parasite infection could have a serious impact on growth and development in children [56]. Djoban and colleagues in Indonesia indicated that, ascariasis causes wasting in children through lactose intolerance, malabsorption of vitamins and other micronutrients, and appetite loss that could impair growth gain. Nevertheless, ascariasis was not associated with wasting in their study [57]. In the current study, 20.5% of children with *Ascaris lumbricoides* infection had wasting, and a significant association was found between *Ascaris lumbricoides* and wasting. Giardiasis is a strong predictor of stunting [58]. In this study, a significant association was found between prevalence of *Giardia lamblia* and stunting. This finding was also consistent with findings from a study by Caron and colleagues in Cambodia, which reported that giardiasis was found to be a protective factor for acute diarrhea, yet, associated with stunting [59]. Our findings suggest that, there is a strong link between nutritional status and parasitic infection [38].

## Limitation

In a few instances, it was not possible to process stool samples within the stipulated time due to distance of study sites from the laboratory, thus it is possible that some motile parasites were lost in the process, and this was a limitation to our study. The proportion of children with low-intensity infections may have been mistakenly categorized as uninfected since investigations for intestinal parasites only used one stool sample from each child which could have led to an underestimation of the prevalence of intestinal parasite infections.

## Conclusion

This study revealed a lower prevalence of intestinal parasites than previously reported and that, malaria and intestinal parasitic infection are significantly associated with anaemia, wasting, stunting, and underweight. These results emphasize the necessity for initiatives of intervention aimed at further lowering the burden of malaria and intestinal parasite infection to reduce their impact on nutritional status of children in the research area. Thus, malaria control initiatives by Ghana Health Service should go hand in hand with effort to improve sanitation especially in areas with poor environments.

## Figures and Tables

**Table 1:** Baseline and demographic characteristics of the studied population

Characteristics	Total number of participants (n= 315)	Percentage (%)
**Study sites**		
PMLC Hospital	113	35.9
Mamprobi Hospital	100	31.7
Shukura Hospital	102	32.4
**Gender**		
Male	133	42
Female	182	58
**Age group**		
6 – 11 months	53	16.8
1 – 3 yrs.	153	48.6
4 – 5 yrs.	109	34.6
**Level of education**		
Not in school	168	53.4
Creche	116	36.8
Kindergarten	31	9.8
**Source of drinking water**		
Sachet water	195	61.9
Bottled water	115	36.5
Piped water	5	1.6
**Signs and symptoms**		
Headache	93	29.5
chills	113	35.9
vomit	109	34.6
**Anti-malaria drugs**		
Yes	170	54
No	145	46
**Anti-helminth drugs (μ)**		
Yes	223	71
No	92	29
**Malaria prevalence**	76	24
*P. falciparum*	59	18.7
*P. malariae*	17	5.3
**Intestinal parasites**	34	10.7
*Giardia lamblia*	17	5.3
*Ascaris lumbricoides*	8	2.5
*Hookworm*	6	1.9
*Trichuris trichiura*	3	0.9
**Prevalence of co-infection**	15	5
prevalence of Anaemia	227	72
Acute anaemia	79	25
Moderate anaemia	126	40
Severe anaemia	22	6.9
**Prevalence of malnutrition**	96	30.4
Acute malnutrition	78	24.7
Moderate malnutrition	12	3.8
Severe malnutrition	6	1.9
stunting	51	16.2
Wasting	77	24.4
Underweight	233	74

n= total number of participants, μ = In the last three months

**Table 2:** Parasitic infection and their relation to socio-demographic.

Variables	Malaria Parasite		Intestinal parasite		Co-infection	

	Positive (%)	Adjusted OR (95 % CI) P value	Positive (%)	Adjusted OR (95%CI) P value	Positive (%)	Adjusted OR (95 % CI) P value

**Gender**		**0.731**		**0.920**		**0.283**
Male	33 (10.4)	0.92 (0.45-1.43) 0.401	11 (3.4)	0.73(0.55-1.63) 0.103	4 (1.2)	0.50 (0.25-2.43) 0.091
Female	43 (13.6)	Ref.	23 (7.3)	Ref.	11 (3.4)	Ref.
**Age group**		**0.391**		**0.313**		**0.151**
6-11 months	12(3.8)	Ref.	4 (1.2)	Ref.	2 (0.6)	Ref.
1-3yrs	37 (11.7)	1.33 (0.45-2.93) 0.097	12 (3.8)	0.43(0.17-2.83) 0.260	9 (2.8)	3.02 (1.17-5.54) 0.147
4-5 years	27 (8.5)	0.73 (0.65-1.33) 0.306	18 (5.7)	3.98(0.68-5.03) 0.610	4 (1.2)	2.16 (1.57-7.59) 0.113
**Symptoms**		**0.104**		**0.079**		**0.301**
Vomiting	47 (14.9)	0.91 (0.65-1.30) 0.251	14 (4.4)	1.64(0.65-4.16) 0.301	8 (2.5)	3.10 (1.82-5.26) 0.410
Headache	22 (6.9)	0.25 (0.17-2.99) 0.093	9 (2.8)	2.25(0.86-5.87) 0.090	5 (1.5)	0.42 (0.17-3.59) 0.093
Chills	53 (16.8)	Ref.	18 (5.7)	Ref	–	Ref
**drinking water**		**0.301**		**0.000** *		**0.173**
Sachet water	47 (14.9)	2.12 (1.57-7.59) 0.207	21 (6.6)	4.19(1.62-8.79) 0.000*	11(3.4)	3.77 (1.32-10.75) 0.314
Piped water	2(0.6)	2.01 (0.17-6.54) 0.170	13 (4.1)	1.79(1.62-4.79) 1.401	4 (1.2)	-
Bottled water	27 (8.5)	Ref	–	Ref	–	Ref
**Malaria prevention**		**0.003** *		**0.309**		**1.045**
Mosquito coil	35 (11.1)	3.44 (0.8, 5.61) 0.010*	19 (6.0)	0.50(0.68-3.20) 0.106	8 (2.5)	0.07(0.02- 3.85) 0.829
Insecticide treated net	29 (9.2)	Ref	13 (4.1)	Ref	7(2.2)	Ref
Insecticide spray	12 (3.8)	0.04 (0.2- 1.58) 0.114	2 (0.6)	0.08(0.01-0.89) 0.291	-	-
**Anti-malaria drug**		**0.214**		**0.206**		**2.042**
Yes	34 (10.7)	0.50 (0.18-3.20) 0.109	4 (1.2)	0.30(0.18-1.90) 0.519	5(1.5)	0.70 (0.01- 3.85) 01.20
No	42 (13.3)	Ref	30 (9.5)	Ref	10(3.1)	Ref
**Anti-helminth drug (μ)**		**1.002**		**0.013** *	-	**0.072**
Yes	39 (12.3)	1.4(0.32-6.14) 0.640	9 (2.8)	0.5(2.32-4.14) 0.000*	6 (1.9)	0.82(0.45- 1.52) 0.107
No	37 (11.7)	Ref	25 (7.9)	Ref	9 (2.8)	Ref

OR = odd ratio, CI = Confidence interval,

* = Significant association, μ= in the last three months

**Table 3:** Logistic Regression analysis showing prevalence of anaemia, malnutrition and their relation with parasitic infection.

Characteristics	Anaemia N(%)	AOR (95%CI) P-value	Malnutrition N (%)	AOR (95%CI) P value
Study site				
PMLC Hosp.	94 (29)	1.40 (0.18–3.10) 0.219	42 (37.1)	2.10 (0.19–4.12) 0.220
Mamprobi Hosp.	76 (24)	0.50 (0.01–1.41) 1.011	24 (21.2)	0.40 (0.11–1.60) 0.091
Shukura Hosp.	57 (18)	0.10 (0.08–4.20) 0.109	30 (26.5)	0.13 (0.04–5.10) 0.209

Gender				
Male	131 (41.5)	2.10 (0.01–5.41) 0.251	39 (12.3)	2.44 (0.4–3.61) 0.218
Female	96 (30.4)	1.10 (0.48–3.10) 0.003*	57 (18.0)	0.30 (0.08–3.40) 0.203

Age group				
6–11 months	(11.7)	0.40 (0.08–3.10) 0.118	29 (12.7)	3.10 (0.41–5.10) 0.003*
1–3 years	(34.9)	1.20 (0.01–3.01) 1.211	53 (23.3)	1.19 (0.04–2.05) 0.213
4–5 years	(25)	0.05 (0.00-4.20) 0.207	14 (6.1)	0.22 (0.03, 1.83) 0.161

Malaria				
*P. falciparum*	50 (65.7)	0.32 (0.11–2.42) 0.000*	23 (30.3)	0.07 (0.01–0.76) 0.426
*P. malariae*	12 (16.3)	0.20 (0.01–3.90) 0.149	4 (5.2)	2.10 (0.48–4.20) 0.313

Intestinal parasites				
*Ascaris lumbricoides*	-	-	8 (23.5)	2.10 (0.18–4.90) 0.259
*Giardia lamblia*	4 (11.7)	0.30 (0.02–2.42) 1.151	3 (8.8)	0.80 (0.12–2.10) 0.000*
Hookworm	5 (14.7)	3.50 (0.01–5.01) 0.021*	-	-
*Trichuris trichiura*	2 (5.8)	1.0 (0.08–4.10) 0.109	1 (2.9)	1.20 (0.48–3.10) 0.221

Co-infection	9 (60)	0.30 (0.02–2.42) 0.151	1 (6.6%)	0.90 (0.48–3.10) 0.403

N= (number of participants), AOR = Adjusted odd ratio,

* = significant association.

**Table 4:** Stunting, wasting and underweight among febrile children in relation to gender, age and parasitic infection.

Variables	Stunting N (%)	P-value	Wasting N (%)	P-value	Underweight N (%)	P-value
Gender						
Male	19 (6)	0.031*	30 (9.5)	0.520	100 (31.7)	1.091
Female	32 (10.2)	0.201	47 (14.9)	1.930	133 (42.2)	0.000*
Age group						
6–11 months	15 (4.7)	0.191	9 (2.8)	0.083	56 (17.7)	0.150
1–3 years	27 (8.7)	0.140	40 (12.6)	2.211	127 (40.3)	0.001*
4–5 years	9 (2.8)	2.019	28 (8.8)	0.910	50 (15.8)	1.130
Malaria						
*P. falciparum*	10 (13.1)	0.130	13 (17.1)	0.412	20 (26.3)	0.013*
*P. malariae*	4 (5.2)	2.110	3 (3.9)	1.061	5 (6.5)	2.010
IPIs						
*Ascaris lumbricoides*	-	-	7 (20.5)	0.010*	3 (8.8)	0.514
*Giardia lamblia*	6 (17.6)	0.000*	-	-	8 (23.5)	0.240
*Hookworm*	3 (8.8)	0.914	4 (11.7)	0.813	-	-
*Trichuris trichiura*	1 (2.9)	1.072	-	-	2 (5.8)	1.003
Co-infection	2 (13.3)	0.941	1 (6.6)	0.106	5 (33.3)	0.502

## Data Availability

The datasets generated and/or analyzed during the current study are available from the corresponding author on reasonable request.

## Abbreviations

IPI: Intestinal Parasite Infection
RBCs: Red blood cells
CHP: Child Health Program
OPD: Outpatient department
PMLH: Princess Marie Louise Children’s hospital
WHO: World Health Organization
HA: Height-for-age
WA: Weight-for-age
WH: Weight-for-height
Hb: Haemoglobin
MUAC: Mid upper arm circumference
